# Notes and News

**Published:** 1903-06-20

**Authors:** 


### NOTES AND NEWS.

THE Queen has made a munificent gift of £5,000 out of her Majesty's War Fund towards the extension of the scheme, initiated by Colonel Sir James Gildea in 1899, for providing self-contained apartments free for the widows and daughters of officers on the same system as those granted by the King at Hampton Court and Kensington Palaces. A suitable site has been obtained at Wimbledon, and building will be proceeded with as sufficient funds are forthcoming. There are at the present time 40 candidates waiting election, in addition to the 12 ladies already temporarily provided for. Contributions can be sent to Colonel Sir James Gildea at 23 Queen Anne's Gate, Westminster, S.W.

THE concert in aid of the Paddington Green Children's Hospital, held on May 25th, secured £600 for the institution.

LITERARY piracy amongst journals is comparatively rare in this country. We are willing, therefore, to believe that provincial journals of the standing of the *Bristol Mercury* and the *Newcastle Leader* would not have knowingly published much of our Hospital Sunday Supplement in their columns without acknowledgment under the head of "Our Great Hospitals: a Record of Beneficent Work." We presume, as the articles are identical and occupy an equal number of columns of space, that they must have been supplied to the *Bristol Mercury* and the *Newcastle Leader* and other papers by some news agency on whom they regularly rely for blocks of copy in stereo. However the piracy may have come about, we confidently look for an apology from the editors of the *Bristol Mercury* and the *Newcastle Leader* now that their attention is called to the matter. If our surmise is correct, we hope that the proprietors of the papers in question will decline to pay for the copy in the circumstances named, for that would be the most effective way of putting a stop to further piracies of this nature.

IN reply to a question asked in the House of Commons on Friday it was stated that as far as statistics were available that of 57,928 persons in India inoculated for the plague 2,740 had died of the disease.

THE Birmingham charities have benefited largely under the will of the late Mr. Thomas Best. The General Hospital and General Dispensary each received £5,000, the Queen's Hospital £3,000, the Children's, the Orthopædic and Women's Hospitals each receive £2,000, and the Eye Hospital and Ear and Throat Hospital £1,000 each.

THE young male nurse who attended Dr. Milan Sachs when suffering from the plague with which he infected himself, and from which he died, is isolated in the Charité Hospital, Berlin, with his volunteer doctor and nurse, Dr. Plugmacher; the only means of communication with others being by telephone. All those who came in contact with Dr. Sachs after he became infected, are isolated in a pavilion guarded by police.

WE understand that a meeting was called this week to further the scheme for moving King's College Hospital to Camberwell. It is stated that an influential and small majority of governors is against removal, but that practically the whole of the medical staff are united in desiring it. We would suggest that a census should be taken of the patients relieved at King's College Hospital during the past year, when we anticipate it will be found that a large number of them came from the district to which it is desired to remove this institution. There can be no doubt that King's College Hospital would prosper in every way were it to be removed from its present site to, say, Camberwell. Indeed, the service which the governors, by consenting to this course, would render to the Metropolis generally, by enabling the resources of King's College Hospital to be utilised for a population of nearly half a million people, who urgently need hospital accommodation, must be recognised by the wealthy, and indeed by all who wish well to the hospitals, and to the metropolis of the Empire.

SIR ERNEST CASSEL having given a large sum of money to the Egyptian Government for the relief of ophthalmia in Egypt, it has been decided to send travelling dispensaries into the country for the relief of those sufferers from eye diseases who are unable to attend the already existing hospitals. There will be at first one of these dispensaries or ambulant hospitals, which will have a couple of tents with beds for operation cases and for the more serious treatment cases. This will travel about from place to place under the direction of an ophthalmic surgeon, who will have under him an Egyptian assistant doctor. If the experiment is successful the number of dispensaries will be increased. Mr. A. F. MacCallan, M.B.Camb., F.R.C.S.Eng., has been appointed by the Egyptian Government to organise and direct the enterprise, with the title of Inspector of Travelling Ophthalmic Dispensaries in Egypt. Mr. MacCallan was formerly senior house-surgeon at the Royal London Ophthalmic Hospital (Moorfields), and at the present time holds office as chief clinical assistant at the hospital. He leaves for Egypt immediately.

